# Probing droplets on superhydrophobic surfaces by synchrotron radiation scattering techniques

**DOI:** 10.1107/S1600577514009849

**Published:** 2014-06-10

**Authors:** Angelo Accardo, Enzo Di Fabrizio, Tania Limongi, Giovanni Marinaro, Christian Riekel

**Affiliations:** a Istituto Italiano di Tecnologia, Via Morego 30, Genova 16163, Italy; b Physical Science and Engineering Divisions, KAUST (King Abdullah University of Science and Technology), Jeddah, Saudi Arabia; c Department of Clinical and Experimental Medicine, BIONEM Lab at University Magna Graecia, Campus Salvatore Venuta, Viale Europa 88100, Germaneto-Catanzaro, Italy; d European Synchrotron Radiation Facility, BP 220, 38043 Grenoble Cedex, France

**Keywords:** superhydrophobic surface, nanotechnology, biological matter, synchrotron radiation micro- and nanodiffraction

## Abstract

Droplets on artificially structured superhydrophobic surfaces represent quasi contact-free sample environments which can be probed by X-ray microbeams and nanobeams in the absence of obstructing walls. This review will discuss basic surface wettability concepts and introduce the technology of structuring surfaces. Quasi contact-free droplets are compared with contact-free droplets; processes related to deposition and evaporation on solid surfaces are discussed. Droplet coalescence based on the electrowetting effect allows the probing of short-time mixing and reaction processes. The review will show for several materials of biological interest that structural processes related to conformational changes, nucleation and assembly during droplet evaporation can be spatially and temporally resolved by raster-scan diffraction techniques. Orientational ordering of anisotropic materials deposited during solidification at pinning sites facilitates the interpretation of structural data.

## Abstract 2

A comprehensive review about the use of micro- and nanostructured superhydrophobic surfaces as a tool for *in situ* X-ray scattering investigations of soft matter and biological materials.

## 1. Introduction   

The upcoming of digital microfluidics has resulted in new technologies for lab-on-chip systems based on the manipulation and analysis of aqueous solution droplets confined by an inert liquid in a microfluidic cell (Song *et al.*, 2006 ▶; Berthier, 2008 ▶; Seemann *et al.*, 2012 ▶). Sample volumes can reach the femtolitre range and less, enabling single-molecule sensitivity in favourable cases (Chiu *et al.*, 2009 ▶). A complementary approach consists of depositing and manipulating droplets on superhydrophobic surfaces (SHSs) providing quasi contact-free conditions (Gentile *et al.*, 2010 ▶; Accardo *et al.*, 2010 ▶, 2011*a* ▶). Evaporation times for droplets on a SHS in air range from tenths of minutes for a few microlitres to a few seconds and less for sub-nanolitre volumes. These times can be modulated by about an order of magnitude to adjust the humidity level (Popov, 2005 ▶), or a quasi-constant droplet volume can be maintained by a drop-on-demand system (Galliker *et al.*, 2013 ▶). Pinning effects during wetting transitions and solidifications result in shear-flow-induced orientational ordering differing from capillary-flow-induced ordering during coffee-ring type solidification (Deegan *et al.*, 1997 ▶) (see §2.2).

This review will focus on X-ray microbeam and nanobeam probing of biological solution droplets on SHSs by wide-angle X-ray scattering (WAXS) and small-angle X-ray scattering (SAXS) techniques. For a complementary review on X-ray probing of droplets on wetting surfaces the reader is referred to Müller-Buschbaum *et al.* (2011 ▶). As compared with electron scattering techniques requiring ultrathin sections, X-ray scattering techniques can be readily used for *in situ* experiments during whole droplet evaporation and for probing residues.

X-ray scattering techniques are routinely used at third-generation synchrotron radiation (SR) sources for high-throughput protein crystallography (Beteva *et al.*, 2006 ▶) or SAXS on protein solutions (Svergun *et al.*, 2013 ▶). The availability of brilliant X-ray microbeams has generated transformative microcrystallography research resulting in particular in advances in amyloid (Nelson *et al.*, 2005 ▶) and membrane protein (Rasmussen *et al.*, 2007 ▶) structures. It is therefore interesting to explore science enabled by X-ray microbeam and nanobeam scattering experiments for droplets with molecules of biological relevance on SHSs, also in view of the emergence of SR sources approaching the diffraction limit such as MAX IV, NSLS II, the ESRF upgrade project and others.

## 2. Methods   

This section will introduce some general features of X-ray microbeam raster-scan probing followed by an overview of the basic concepts related to surface wettability and the typical fabrication steps of the devices used in the X-ray experiments.

### 2.1. X-ray raster-scan probing   

The heterogeneity of soft and biological matter can be probed by X-ray raster-scan techniques. Beam sizes of ∼1 µm at λ ≃ 0.1 nm wavelength are routinely available at several ESRF beamlines and other SR sources worldwide while intense X-ray nanobeams down to the 100 nm range and smaller have become available more recently (Riekel *et al.*, 2009 ▶; Weinhausen *et al.*, 2012 ▶). Raster-step increments are usually chosen to be larger than the beam size in order to avoid radiation damage propagating into neighbouring scan-points.

Depending on the interaction channel of X-rays with matter, probes can make use of different contrast modes (absorption, fluorescence,…). The present review will be mostly limited to the elastic X-ray scattering contrast used in SAXS/WAXS probes which are sensitive to electron density fluctuations (Δρ) at different length scales. Indeed, WAXS techniques probe microstructure, crystallinity, particle size or texture at the unit-cell level while SAXS techniques probe Δρ at the mesoscale for non-periodic (single particles) or periodic objects (*e.g.* semi-crystalline lattices) (Guinier & Fournet, 1955 ▶). MicroSAXS/WAXS (µSAXS/WAXS) techniques are therefore sensitive to the hierarchical organization of matter, extending to macroscopic scales when combined with raster-scan techniques (Riekel *et al.*, 2009 ▶). It is often sufficient to map characteristic scattering features across a sample in order to reveal fingerprints of specific microstructures or morphologies. Complementary information can be obtained by combining raster µSAXS/WAXS probes with other probes such as Fourier transform infrared spectroscopy (FTIR) (see §3), optical [*e.g.* ellipsometry (Roth *et al.*, 2011 ▶)] or Raman (Davies *et al.*, 2008 ▶).

Experiments in transmission geometry are performed with the X-ray focal spot at the sample position (Riekel *et al.*, 2009 ▶). Grazing-incidence small-angle X-ray scattering (GISAXS) can probe surface-sensitive features at the expense of an enlargement of the footprint along the beam direction (Müller-Buschbaum, 2003 ▶; Roth *et al.*, 2003 ▶; Gebhardt *et al.*, 2009 ▶).

### 2.2. Basic wettability concepts   

**Figure 1:**
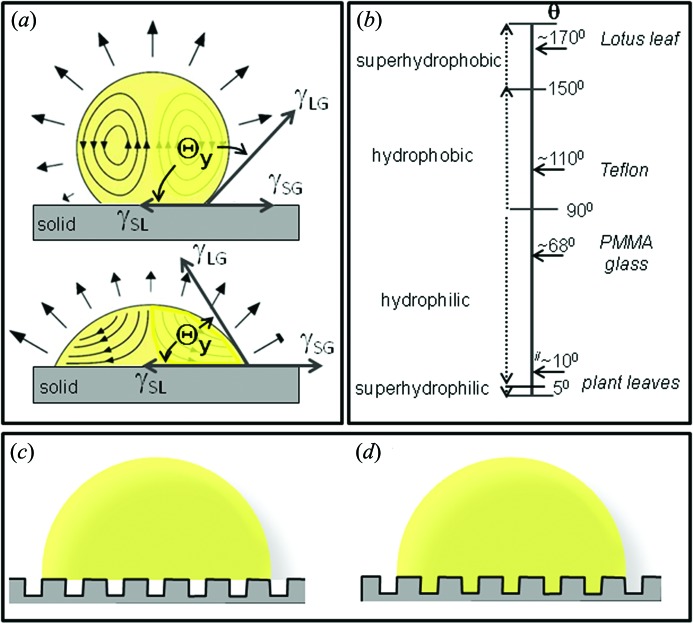
(*a*) Equilibrium states of droplets for a hydrophobic (top) and a hydrophilic (bottom) surface. The convective flows induced by evaporation, interfacial surface energies and the contact angle are shown. [Adapted from Ressine *et al.* (2008 ▶).] (*b*) Classification of surfaces according to their contact angles Θ. Note that superhydrophobic PMMA (PMMA^sphob^) relies on a thin hydrophobic Teflon layer. (*c*) Unpinned Cassie–Baxter (‘Fakir’) state and (*d*) pinned Wenzel (‘spread’) state on a SHS with asperities (*e.g.* micropillars; see §3.2).

The wettability of a flat surface can be expressed by its contact angle (CA), Θ, at the liquid/gas interface of a droplet [Figs. 1(*a*) and 1(*b*) ▶]. The CA value is expressed in Young’s equation as resulting from the thermodynamic equilibrium of the free energy at the solid/liquid/gas interphase (Fig. 1*a* ▶),
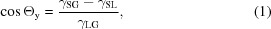
where γ_SG_, γ_SL_ and γ_LG_ are, respectively, the interfacial surface energies between solid (S), liquid (L) and gas (G) phases.

While for a smooth surface the equilibrium of the surface energies is given by Young’s equation (1), that for rough surfaces is defined according to Wenzel (1936 ▶) as
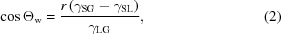
where *r* is the ratio between the actual interface and the geometric interface corresponding to the projected surface, and Θ_w_ and Θ_y_ indicate the CAs of the Wenzel and Young models, respectively, which are related by
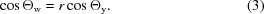
SHSs are usually constituted of micro- and/or nano-asperities and the droplet can assume two different ‘states’: (i) penetrating the asperities in a pinned (‘spread’) state or (ii) remaining on top of the asperities in a suspended (‘Fakir’) state [Figs. 1(*c*) and 1(*d*) ▶]. These two states are described by the Wenzel (1936 ▶) and Cassie–Baxter (Cassie & Baxter, 1944 ▶) equations. The Wenzel model assumes that the liquid adapts to the surface roughness and, at thermodynamic equilibrium, there is a linear relationship between the CA on the rough surface and the so-called ‘roughness factor’ *r* in equation (3). Θ_w_ corresponds here to the CA on the rough surface and Θ_y_ is the CA relative to a flat surface made of the same material. For a rough surface, therefore, *r* > 1 holds. Indeed, for a hydrophobic surface, Θ_w_ > Θ > 90°, and, for a hydrophilic one, Θ_w_ < Θ < 90° (Fig. 1*b* ▶). This implies that the presence of surface roughness can drive a hydrophobic surface into the superhydrophobic state and a hydrophilic surface into the superhydrophilic state.

It is important to note that the local evaporation rate depends on the CA. Indeed, for droplets on wetting surfaces, the evaporation rate will be highest at the triple contact-line resulting in an outward convective flow, pinning and the formation of a coffee-ring residue (Deegan *et al.*, 1997 ▶). In contrast, droplets on SHSs have a more homogeneous evaporation rate across its surface resulting in the presence of a circulatory convective flow (Fig. 1*a* ▶). For a solid surface with an area fraction ϕ and an intrinsic CA, Θ_e_, the freely suspended fraction containing air corresponds to (1 − ϕ). The CA is defined according to the Cassie–Baxter law (Cassie & Baxter, 1944 ▶),
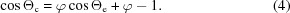
The Cassie–Baxter law is frequently used to describe SHS behaviour with CAs above 150° (Fig. 1*b* ▶). Considering a small displacement of the contact line of a droplet during evaporation, the suspended Cassie–Baxter state is thermodynamically stable if the change in surface energy per unit length associated with this displacement is smaller than the state leading to the Wenzel state (Bico *et al.*, 2002 ▶). Hence, the condition of stability for this state is
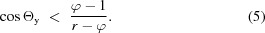
Indeed, the solid substrate must be sufficiently hydrophobic for air pockets to be stable. For a Young’s CA between 90° and the threshold value given by the previous equation, the air pockets will be metastable.

Both Wenzel and Cassie–Baxter equations provide a quantitative prediction of roughness effects which can be experimentally verified by CA measurements. Indeed, for an appropriate design of a SHS, an appropriate model predicting wetting behaviour is fundamental. According to the previous threshold condition, a water droplet on a SHS can transit from a Cassie–Baxter to a Wenzel state (called ‘wetting transition’). Both states correspond to local energy minima of the system and are therefore stable states associated with discrete energy levels. Indeed, the system can switch between stable states due to small perturbations. The wetting transition into the state with the lowest CA, corresponding to a global energy minimum, is associated with an energy barrier which depends on the surface features (Giacomello *et al.*, 2012 ▶). It is also directly correlated to the evaporation dynamics of a droplet which depends on its state [Figs. 1(*c*) and 1(*d*) ▶]. Indeed, one can consider three cases:

(i) the droplet remains pinned and the CA is reduced during the evaporation;

(ii) the droplet is not pinned so that the CA does not or only slightly changes;

(iii) the droplet is not pinned until a certain volume is reached and then starts to become pinned with a reduction of its CA.

While case (ii) is observed for pure water or for low solute concentration droplets, case (iii) is observed for droplets for which the evaporation-induced increase of solute concentration results in pinning at its rim.

**Figure 2:**
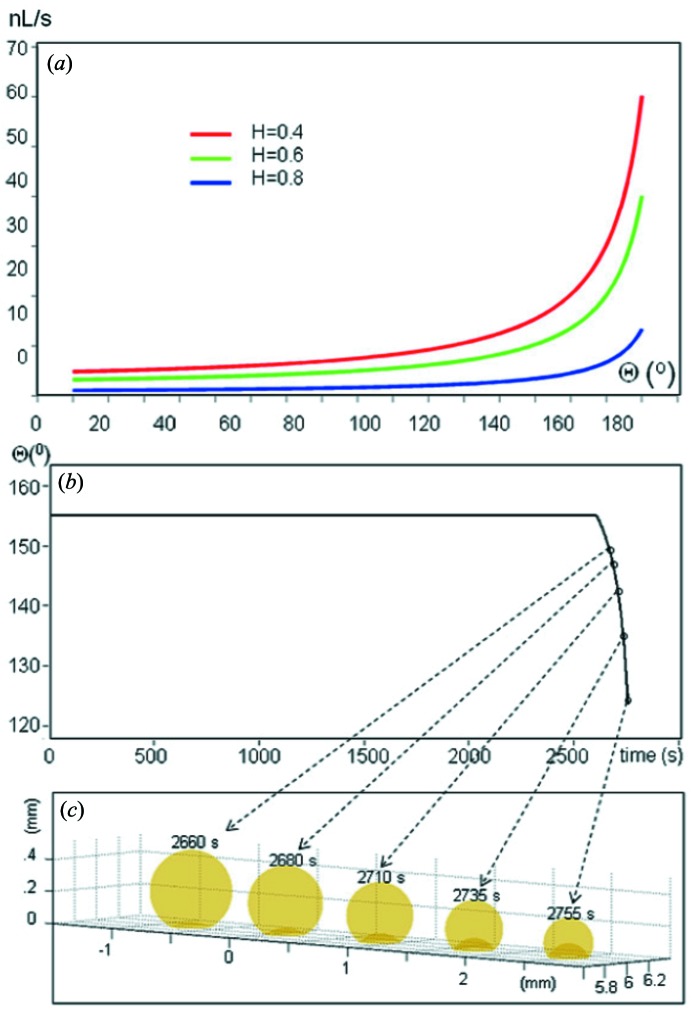
(*a*) Simulation of evaporation rate (nL s^−1^) of a 5 µL sessile droplet as a function of the CA (Θ) based on equation (6) (Marinaro, 2013 ▶). The curve of the evaporation rate over the CA is proportional to (1 − *H*), where *H* is the humidity. (*b*) Simulation of the CA change of a 5 µL droplet during evaporation at room temperature. The decrease of CA for *t* > 2600 s is due to the wetting transition. (*c*) Simulation of droplet shape change during wetting transition. Note the development of a hollow shape (Marinaro, 2013 ▶).

It is possible to predict the evaporation rate, corresponding to the rate of mass loss (d*M*/d*t*), of a droplet for the three cases based on diffusion-based models developed for all possible CAs (Popov, 2005 ▶) in good agreement with experimental results. Indeed, the model for the largest range of CAs has been derived by Popov (2005 ▶),
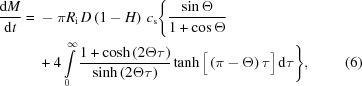
where τ is the dimensionless time (Popov, 2005 ▶), *H* is the humidity, *D* the vapour diffusivity, *R*_i_ the droplet radius at the interface, *c*_s_ the saturated vapour concentration and Θ the CA. An analytical solution based on the Laplace equation has been obtained by Lebedev (1965 ▶). The evaporation rate derived from equation (6) (Marinaro, 2013 ▶) reveals a strong influence of the humidity on the evaporation rate (Fig. 2*a* ▶) which can be used for influencing the assembly rate of macromolecules at the liquid/air and liquid/solid interfaces.

Based on equation (6) it is also possible to model the evolution of a droplet during the wetting transition on a typical SHS composed of a pattern of micropillars (presented in §2.3) which allows the formation of air pockets under the droplet in a suspended ‘fakir’ state [Fig. 1(*c*) ▶]. We will consider the case of a 5 µL droplet with initial CA = 155° and *H* = 60% for the first 1300 s of evaporation followed by the wetting transition [for experimental CA measurements, see Accardo (2012 ▶)]. During evaporation, the droplet radius and evaporation rate decreases as the contact surface and the contact angle are imposed to be constant (Fig. 2*b* ▶). In practice one observes at the wetting transition for high molecular concentrations often the formation of hollow residues [*e.g.* Fig. 6(*e*)]. The evaporation rate is evaluated for this morphology as the sum of two components: the external evaporation rate (along the outer surface) and the evaporation rate below the interface between the droplet and the surface. The formation of the hollow is therefore treated as a further evaporation process where the air between the micropillars creates a concentration gradient of vapour allowing the vapour to diffuse and modify the interface. The simulation takes into account the spherical cap approximation of the droplet shape and assumes a constant CA until a nominal concentration value is reached. The pinning state and the beginning of formation of the hollow are introduced at the same instant. The shape evolution at selected points of the wetting curve is shown in Fig. 2(*c*) ▶.

### 2.3. Fabrication of SHSs   

**Figure 3:**
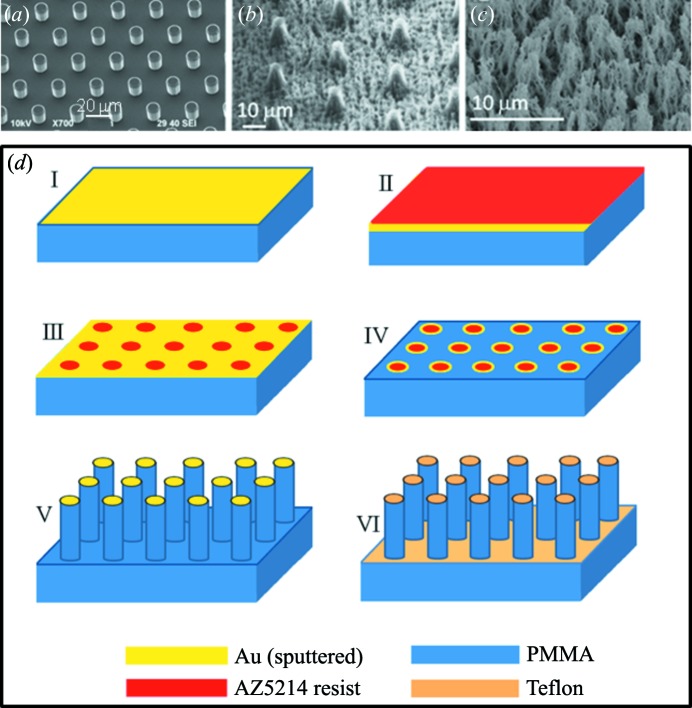
Scanning electron microscopy (SEM) images of SHSs showing microstructural (micropillars) and nanostructural (nanofibrils) features. (*a*) Nanopatterned pillared silicon surface. (*b*) PMMA surface with a hierarchical roughness composed of micropillars and nanofibrils. (*c*) Nanofibrillar PMMA surface. [Adapted from Limongi *et al.* (2013 ▶) and Accardo *et al.* (2010 ▶).] (*d*) Micro-fabrication process steps to develop micropatterned superhydrophobic PMMA surface. (I) Gold sputtering; (II) AZ5214 spin-coating; (III) resist baking, exposure, tone-inversion and development; (IV) gold etch; (V) DRIE plasma process; (VI) gold stripping and Teflon coating. [Adapted from Accardo *et al.* (2010 ▶).]

This section will provide an overview on the fabrications of selected SHSs such as ‘passive’ SHSs based on a polymethylmethacrylate (PMMA) substrate which are optically transparent and have a lower X-ray absorption coefficient than silicon. For technological details on micropillared superhydrophobic silicon substrates (Fig. 3*a* ▶) the reader is referred to De Angelis *et al.* (2011 ▶). Repetitive patterns can be readily extended to the nanoscale. Indeed, by using a thin patterned polymeric film, ≥10 nm features with spacings of ≥100 nm can be etched into silicon (Checco *et al.*, 2014 ▶). The final section will cover ‘active’ superhydrophobic chips making use of the electrowetting principle.

#### 2.3.1. Passive SHSs   

Several SHSs based on silicon micropillars, PMMA micropillars, and nanofibrils and PMMA nanofibrils are shown in Figs. 3(*a*)–3(*c*) ▶. The hierarchically organized surface feature in Fig. 3(*b*) ▶ resembles remarkably morphological features of lotus leaves with similar CAs up to ∼170° (Fig. 1*b*). Nanofibrillar superhydrophobic PMMA (Fig. 3*c* ▶) has a comparable CA (Accardo *et al.*, 2010 ▶).

The fabrication process of a micro- and nano-structured PMMA surface (Fig. 3*b* ▶) is shown schematically in Fig. 3(*d*) ▶ (Accardo *et al.*, 2010 ▶). PMMA sheets are coated with a 100 nm gold layer by a sputtering process. A layer of positive tone resist is spin-coated (II) and baked. A proximity mask with the pillar pattern is then exposed to UV (λ ≃ 365 nm). After baking, a further UV exposure without the optical mask makes the previously unexposed areas soluble to a development step which creates the resist array pattern. (III) To remove the exposed gold areas an isotropic wet etch based on aqueous KI/I_2_ solution is used. (IV) The final step is composed of a two-step plasma process spaced out by a gold etch removal. An O_2_/Ar gas mixture is used to define the pillar-like structure allowing both an ion-enhanced chemical and a physical sputter etching to be carried out. The Au mask caps are removed from the top of the micropillars by a wet etch process (V) and a C_4_F_8_ plasma process is used to cover the whole surface with a thin (5–10 nm) Teflon layer (VI). Avoiding the optical lithography step allows a significant speed-up of the whole process. Indeed, a nanofibrillar PMMA surface is obtained through a two-step plasma-process (pure oxygen for the texturing and C_4_F_8_ for the Teflon layer) with a processing time of only 12 min. This assumes a high relevance in terms of throughput as the plasma process allows several surfaces to be processed simultaneously (Accardo *et al.*, 2010 ▶) (Fig. 3*c* ▶).

**Figure 4:**
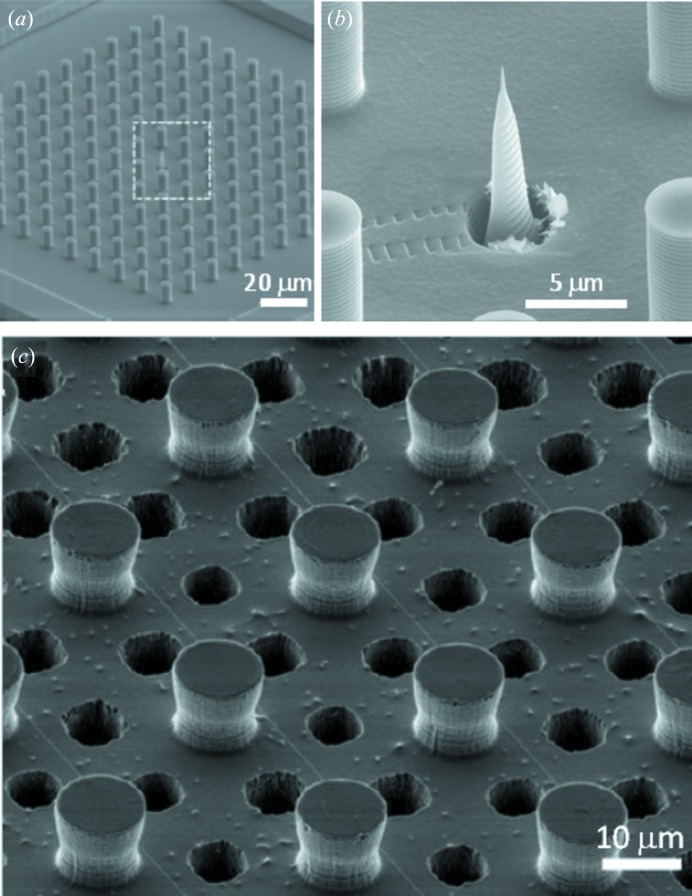
(*a*) Superhydrophobic silicon chip with a forest of micropillars surrounding a microcone (De Angelis *et al.*, 2011 ▶). (*b*) Details of the microcone with embedded plasmonic device. [Adapted from De Angelis *et al.* (2011 ▶).] (*c*) Micropillared superhydrophobic silicon surface with holes between the micropillars and drawn λ-DNA nanofilaments. [Adapted from Gentile *et al.* (2012 ▶).]

While nanofibrillar PMMA surfaces can be produced more economically, droplets on microstructured SHSs can be easier immobilized in a stable position for probing experiments. The time required for aligning a droplet in a SR microbeam is generally not an important issue for experiments with microlitre droplets involving evaporation times exceeding several tenths of minutes. The deposition of small-volume droplets with shorter evaporation times requires, however, a different approach. Indeed, a radial gradient in pillar-spacing provides an attraction point for a deposited droplet (Gentile *et al.*, 2013 ▶). An elegant solution is also provided by introducing a defect, such as a Si microcone produced by focused ion-beam milling in a forest of Si micropillars, serving as an attraction point for droplets during deposition (De Angelis *et al.*, 2011 ▶) [Figs. 4(*a*) and 4(*b*) ▶].

A further special pillared Si SHS is shown in Fig. 4(*c*) ▶. The holes drilled by a deep reactive etching (DRIE) process between the micropillars allow the transmission of electrons and X-rays without absorption by the substrate. This is of interest for probing very small quantities of biomaterials deposited on the micropillars such as λ-DNA nanofilaments. The fabrication process requires double polished silicon wafers (both-sided photolithography) of 50 µm diameter for the DRIE process (Gentile *et al.*, 2012 ▶). The main challenge in the fabrication process is the alignment procedure of the holes between the micropillars pattern.

#### 2.3.2. Active SHSs   

**Figure 5:**
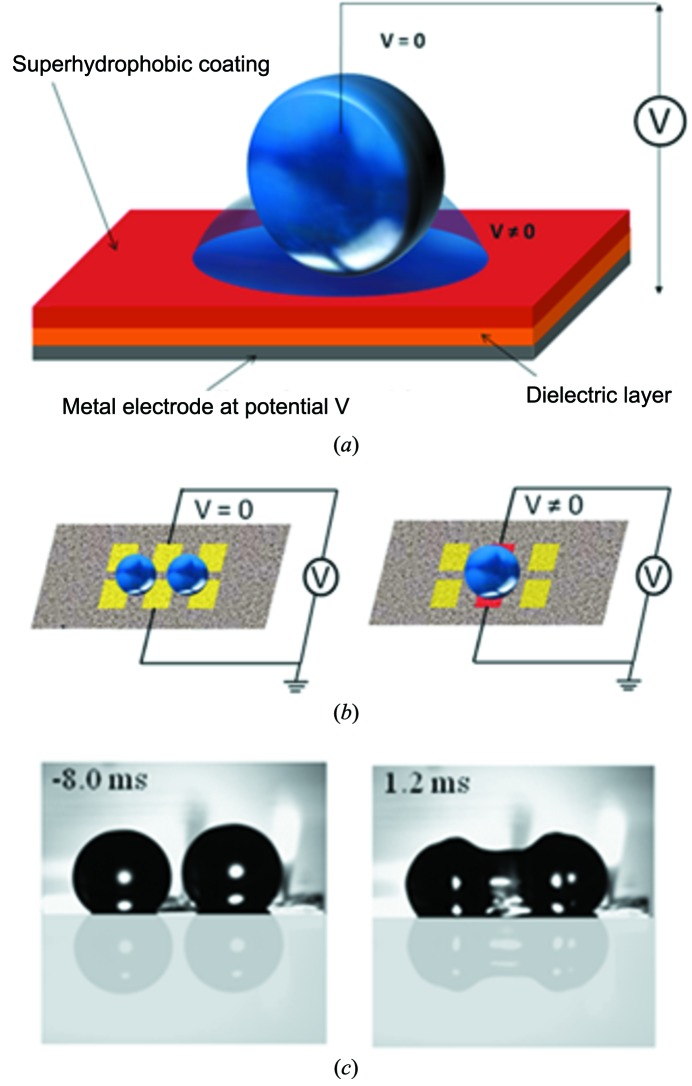
(*a*) Principle of the EWOD device with a SHS (SHEWOD) showing a droplet in the high contact angle (suspended) state (*V* = 0). Once the voltage (*V* ≠ 0) is applied, the contact angle decreases and the droplet spreads. (*b*) Multiple electrode SHEWOD device showing schematically the coalescence of two droplets. (*c*) Selected 200 µs video frames from a CMOS camera during coalescence of two ∼4 µL water droplets on the SHEWOD device at 45 V AC (1 kHz). [Adapted from Accardo *et al.* (2013*a* ▶).]

The development of ‘active’ SHS chips allows *in situ* probing of droplet mixing, avoiding largely wall effects (Accardo *et al.*, 2013*a* ▶). The principle is shown schematically in Fig. 5(*a*) ▶ for a droplet on an electrowetting on dielectrics (EWOD) device with an embedded SHS. Voltage tuning implies changing the CA from Θ ≃ 160° (superhydrophobic) to Θ ≃ 110° (hydrophobic) due to the top Teflon layer. This principle can be exploited for droplet mixing driven by the balance of inertia and surface tension in a SHEWOD (superhydrophobic EWOD) device integrating a planar electrode structure in a SHS (Accardo *et al.*, 2013*a* ▶) (Fig. 5*b* ▶). A fast-framing CMOS camera reveals, for the head-to-head coalescence of two ∼4 µL water drops, that the interfaces advance at a speed of ∼15 mm s^−1^ starting the coalescence process with the growth of a liquid bridge (Accardo *et al.*, 2013*a* ▶) (Fig. 5*c* ▶).

The SHEWOD devices combine the advantage of an open planar geometry with high droplet mobility. The multi-electrode platform is a novel approach with respect to single-electrode SHEWOD systems. The device is based on a Si surface equipped with metal electrodes deposited by a plasma vapour deposition process then coated with a 200 nm SiO_2_ layer and a 1 µm-thick PMMA stratum. A nanofibrillar PMMA layer produces the superhydrophobic properties (Accardo, 2012 ▶) which are confirmed by a CA of water droplets of 171.3°.

### 2.4. Quasi contact-free and contact-free droplets   

Quasi contact-free aqueous droplets on SHSs differ in several aspects from aqueous contact-free droplets supported by acoustic levitation (Welter & Neidhart, 1997 ▶), acoustic ejection (Soares *et al.*, 2011 ▶) and drop-on demand systems based on acoustic (Roessler *et al.*, 2013 ▶), piezoconstriction and other ejection modes (Lee, 2003 ▶). Indeed, droplets on SHSs are rotationally immobile allowing probing locally of interface assembly or nucleation by a microbeam (Accardo *et al.*, 2011*b* ▶) in contrast to acoustically levitated droplets (Wolf *et al.*, 2008 ▶).

Stroboscopic µSAXS experiments based on inkjet systems using picolitre-volume droplets of a few m s^−1^ speed allow also probing the interface but only for a droplet lifetime of a few milliseconds (Graceffa *et al.*, 2009 ▶). Single droplet experiments allowing the study of random or chaotic processes in dynamical systems are in principle feasible using femtosecond XFEL flashes and possibly ≤microsecond flashes at SR sources approaching the diffraction limit. The dominating droplet surface energy of picolitre-volume droplets enables also highly localized surface deposition (Schoeck *et al.*, 2007 ▶; Lemke *et al.*, 2004 ▶).

The coalescence regime (Gotaas *et al.*, 2007 ▶) is accessible to droplets on SHSs (Accardo *et al.*, 2013*a* ▶) and from inkjet systems (Graceffa *et al.*, 2012 ▶). The liquid bridge formation at the onset of droplet coalescence (Fig. 5*c* ▶) has been studied in the inviscid regime for undistorted hemispherical droplets generated by capillary nozzles (Case & Nagel, 2008 ▶). The upper limit of droplet distortion is assumed to be at a Weber number of We = 1.1 [We = 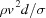 where ρ is the density, *d* is the droplet diameter, σ is the surface tension and *v* is the velocity (Duan *et al.*, 2003 ▶)]. Picolitre-volume droplets generated by inkjets with We ≃ 3.2 (Graceffa *et al.*, 2012 ▶) are already beyond this limit while microlitre-volume droplets on a SHEWOD device with We ≃ 10^−3^ (Accardo *et al.*, 2013*a* ▶) are practically undistorted suggesting that fluid simulations on the onset of coalescence based on Navier–Stokes equations (Eggers *et al.*, 1999 ▶) could be used. It should also be possible to develop multielectrode SHEWOD devices allowing the coordinated coalescence of several droplets, which is of interest to aerosol and cloud physics.

In practice, quasi contact-free droplets on SHSs provide a flexible and cost-effective approach with respect to contact-free droplet environments. Flat SHSs have to be well aligned in order to avoid droplet movements, in particular for raster-scan data collection. The substrate will shadow the lower part of scattering patterns emanating from droplets or residues. This is particularly the case for more absorbing Si substrates while light-atom substrates, such as PMMA, allow in principle sample scattering to be extracted from substrate-scattering (Accardo *et al.*, 2010 ▶). The lack of X-ray scattering from walls and surrounding liquids increases the sensitivity for weak scattering contributions from the droplets, *e.g.* during nucleation events, as compared with microfluidic environments using glass or polymeric windows. The highly homogeneous droplet evaporation in the non-wetting (Cassie–Baxter) regime (see above) avoids coffee-ring effects which are due to an enhanced evaporation at the triple contact-line of droplets on wetting surfaces (Deegan *et al.*, 1997 ▶). Surface-pinning effects are only observed at an advanced stage of evaporation on a SHS at the wetting transition. This allows probing for volume and surface nucleation effects which are practically not influenced by interactions with solid surfaces.

### 2.5. Droplet deposition and residue formation   

**Figure 6:**
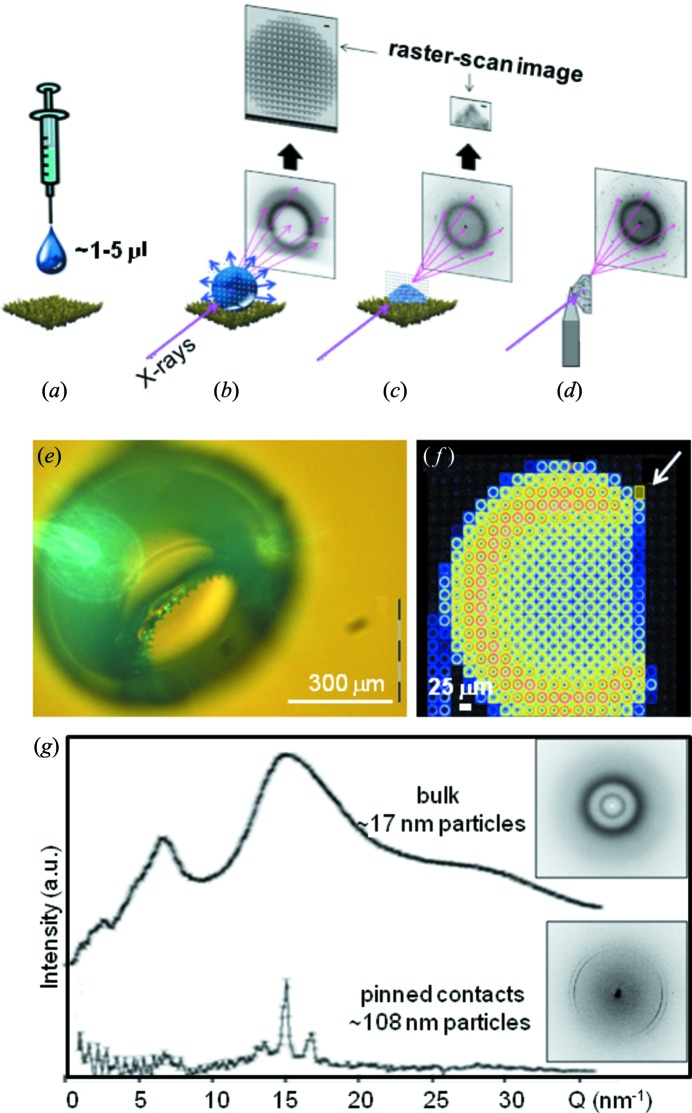
(*a*) Deposition of a solution droplet by a syringe on a nanofibrillar SHS. (*b*) Consecutive raster-diffraction scans of the droplet during evaporation. (*c*) Raster-diffraction of the residue. (*d*) Optional transfer and attachment of the residue to a glass tip and raster-scan. [Adapted from Accardo *et al.* (2011*b* ▶).] (*e*) Hollow lysozyme residue from a SHS (Accardo *et al.*, 2010 ▶). (*f*) Raster-scan image of hollow lysozyme residue with 25 µm step resolution (Accardo *et al.*, 2010 ▶). The arrow indicates the position of a pinned contact. (*g*) Azimuthally averaged diffraction pattern from the core of the residue (top) and from the pinned contact (bottom). Individual diffraction patterns from the two zones are shown to the right. [Adapted from Accardo *et al.* (2010 ▶).]

Droplets in the range of several microlitres are deposited by a syringe on a SHS (Accardo *et al.*, 2011*b* ▶) [Figs. 6(*a*)–6(*d*) ▶]. Probing a droplet *in situ* at selected evaporation times allows raster-scan images to be assembled, composed of ‘pixels’ of individual SAXS/WAXS patterns corresponding to a projection of the volume-scattering onto a plane (Fig. 6*b* ▶). The residue can also be raster-scanned (Fig. 6*c* ▶) or optionally detached from the surface, glued to a glass tip, and further analyzed by raster-scans combined with sample rotation to reveal preferred orientation effects (Fig. 6*d* ▶). Droplet volumes in the nanolitre to picolitre range and lower have to be deposited by an adapted microdrop or nanodrop system.

Pinning effects due to viscous attachments of the evaporating droplet to the SHS are at the origin of the formation of hollow residue morphologies observed at high solute concentrations as shown for lysozyme in Figs. 6(*e*) and 6(*f*) ▶ (Accardo *et al.*, 2010 ▶). We note the good correspondence of the modelling shown in Figs. 2(*b*) and 2(*c*) ▶. Diffraction patterns from the pinned contacts reveal a higher orientation and crystallinity as well as a larger particle size as compared with the bulk of the residue (Accardo *et al.*, 2010 ▶) (Fig. 6*g* ▶). Rim formation and the collapse of a thin shell is observed at low solute concentrations (Accardo, 2012 ▶). Colloidal nanoparticle ordering at the contact line of an evaporating droplet on a wetting surface can be probed by GISAXS (Roth *et al.*, 2007 ▶, 2010 ▶). Ordering effects during collapse of the shell formed on top of a drying latex droplet have been revealed by ultrasmall-angle X-ray scattering (Chen *et al.*, 2012 ▶). Similar processes presumably occur in the final stage of evaporation of low solute concentration droplets on a pillared SHS.

For ultradilute droplets of λ-DNA, the formation of nanofilaments at the rim of the residue is attributed to a mixture of shearing and capillary forces (De Angelis *et al.*, 2011 ▶). SHSs with features breaking the repetitive pillar-pattern, such as a central cone or a gradient, allow compacting molecules from ultradilute droplets at defined pinning sites (De Angelis *et al.*, 2011 ▶; Gentile *et al.*, 2013 ▶).

## 3. Applications   

This section will provide an overview on selected experiments on samples with biological relevance such as CaCO_3_ mineralization and amyloidic aggregation. The presence of conformational mixtures in β-type materials can be probed by a combination of µSAXS/WAXS and µFTIR. The techniques developed for smaller molecules can also be applied to complex biological objects such as cells and subcellular components.

### 3.1. CaCO_3_ mineralization   

**Figure 7:**
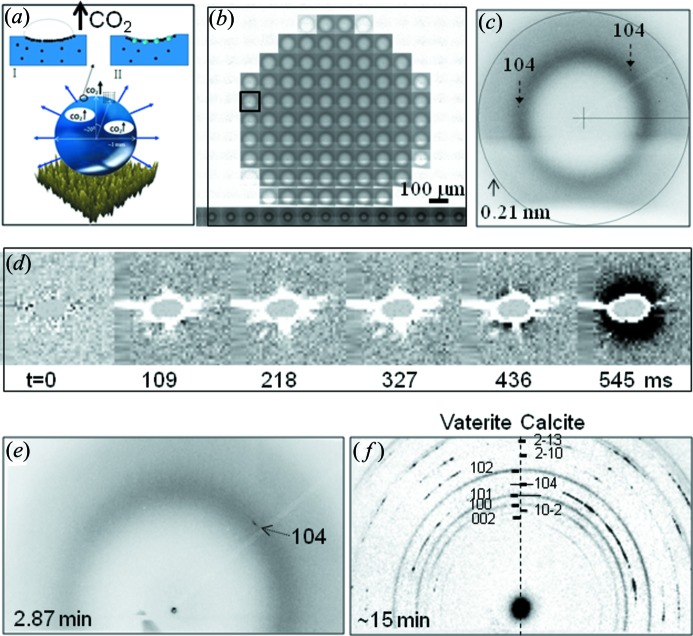
(*a*) Decomposition of Ca(HCO_3_)_2_ solution into CaCO_3_ patches and CO_2_ at the interface of an evaporating droplet (Accardo *et al.*, 2011*b* ▶). The raster-scan across the interface is schematically indicated. (*b*) WAXS raster-scan image of a whole droplet during evaporation. (*c*) WAXS pattern from the interface [rectangle in (*b*)] revealing two calcite crystallites *via* their 104 reflections. [Adapted from Accardo *et al.* (2011*b* ▶).] (*d*) Time series of 100 ms µSAXS patterns during reactive mixing of CaCl_2_ and Na_2_CO_3_ recorded by a pixel detector. The time after the onset of the reaction (*t* = 0) when the corresponding pattern was collected and written to disk is indicated. Negative intensities around the beamstop (in white) are due to beam absorption by the merged droplets as the *t* = 0 pattern with the beam passing between the two droplets has been subtracted from subsequent patterns. SAXS intensity (in black) due to ACC particles is observed at *t* = 436 ms. (*e*) WAXS patterns at 2.87 min revealing a calcite crystallite *via* its 104 reflection. (*f*) Textured powder diffraction from residue due to calcite and vaterite phases. [Adapted from Accardo *et al.* (2013*a* ▶).]

Probing CaCO_3_ formation without the influence of sample cell walls on the reaction products is of interest for research on biomineralization. Indeed, the evaporation of a 4 µL Ca(HCO_3_)_2_ solution droplet on a superhydrophobic PMMA surface reveals the nucleation of calcite crystallites at the solid–liquid interface while the less stable vaterite modification is only observed in the residue (Accardo *et al.*, 2011*b* ▶) [Fig. 7(*a*)–7(*c*) ▶]. The nucleation event was probed by consecutive µWAXS raster-scans of the retreating interface (Fig. 7*a* ▶).

Probing the formation of CaCO_3_ by mixing droplets of CaCl_2_ and Na_2_CO_3_ solutions *via* the SHEWOD device (Fig. 5*b* ▶) and using a fast pixel detector allows ∼100 ms timescales to be accessed (Accardo *et al.*, 2013*a* ▶) [Figs. 7(*d*)–7(*f*) ▶]. The reaction was probed by SAXS/WAXS, placing the ∼1 µm beam prior to coalescence in the middle between the two droplets (Accardo *et al.*, 2013*a* ▶) (Fig. 5*c* ▶). The appearance of SAXS intensity around the beamstop at about 400 ms after the onset of droplet mixing (Fig. 7*d* ▶) agrees with the timescale of amorphous calcium carbonate (ACC) particle formation deduced from stopped-flow SAXS experiments (Bolze *et al.*, 2002 ▶). Although a slight SAXS intensity increase was observed already within ∼200 ms, a clear signature of density fluctuations, deduced by transmission electron microscopy on samples flash-frozen within 100 ms reaction time (Rieger *et al.*, 2007 ▶), could not be obtained. Calcite crystallites were again observed as first crystalline phase while vaterite was only observed in the residue [Figs. 7(*e*) and 7(*f*) ▶].

The ongoing increase in SR source brilliance and the availability of fast pixel detectors suggests, however, the possibility of SAXS data collection on the <100 ms timescale avoiding flash-freezing approaches (Rieger *et al.*, 2007 ▶). In addition, phase-contrast imaging (Fezzaa & Wang, 2008 ▶) could provide complementary information on the emergence of mesoscale inhomogeneities during CaCO_3_ precipitation (Rieger *et al.*, 2007 ▶). Scattering and imaging experiments extending into the microsecond range and smaller will, however, require single X-ray flashes from SR (Ihee *et al.*, 2005 ▶) or XFEL sources (Chapman *et al.*, 2011 ▶). This might also provide a glimpse into the fascinating world of non-equilibrium thermodynamics for strong diffusion gradients in confined volumes (Reguera *et al.*, 2005 ▶).

### 3.2. Biological materials   

#### 3.2.1. Amyloidal aggregates   

**Figure 8:**
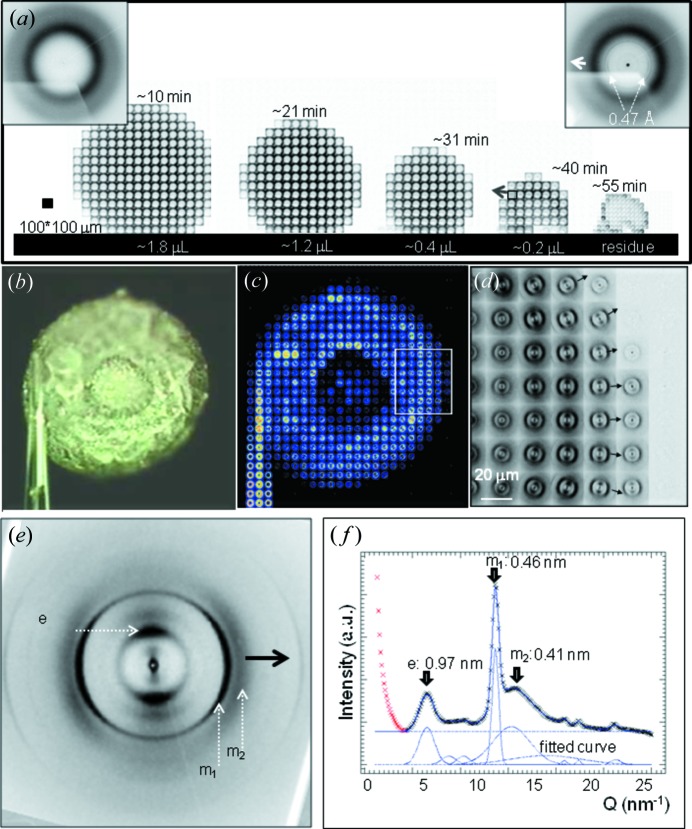
(*a*) Ac-IVD solution droplet evaporating on a superhydrophobic PMMA surface. The droplet was raster-scanned at specific times using a 1 µm SR beam (Hauser *et al.*, 2011 ▶). Nanofibrillation is observed at the droplet interface (grey square) *via* a 0.47 nm cross-β-type peak at *t* ≃ 40 min after droplet deposition. The white arrow indicates the orientation of the fibre axis. (*b*) Optical image of hollow residue attached to glass capillary. (*c*) Raster-diffraction image of hollow residue. (*d*) Zoom into the diffraction patterns from within the rectangular zone in (*c*). The radial orientation of the cross-β fibre axes is indicated by the arrows. (*e*) Selected cross-β diffraction pattern. The strongest peaks along the meridional (m) fibre axis (arrow) and equatorial (e) directions are indicated. (*f*) Intensity distribution along the fibre axis fitted by six Bragg and two short-range-order peaks. The peaks are indicated in (*e*). [Adapted from Hauser *et al.* (2011 ▶).] The Bragg peaks can be indexed for a 5.72 nm cross-β period.

Amyloids are insoluble fibrous extracellular protein deposits which have been related to more than 20 human diseases (Kumar *et al.*, 2009 ▶). The cross-β structure of the amyloidic core structure has been determined by X-ray microdiffraction for peptide microcrystals (Nelson *et al.*, 2005 ▶). Fibrillation can also be studied for peptide model systems. Indeed, hydrogel-forming short tri- to hexapeptides have been shown forming β-type materials by circular dichroism spectroscopy. The random orientation of the nanofibrillar material did not allow more detailed X-ray studies. Highly oriented X-ray fibre diffraction patterns have, however, been obtained for peptide solution droplets drying on a superhydrophobic PMMA surface as shown in Figs. 8(*a*)–8(*f*) ▶ for Ac-IVD (Hauser *et al.*, 2011 ▶). Fibrillation was observed starting at about 40 min after droplet deposition as revealed by a β-type 0.47 nm reflection appearing at the interface of the droplet (Fig. 8*a* ▶). The hollow residue (Fig. 8*b* ▶) shows a high fibrillar orientation at the pinning points towards the interface due to shearing effects during pinning [Figs. 8(*c*)–8(*f*) ▶]. Similar orientation observations were made for Ac-LIVAGD and amylin (Lakshmanan *et al.*, 2013 ▶). Lysozyme protein in the presence of high Ca^2+^ concentrations also shows amyloidic fibrillation in droplets on SHSs (Accardo *et al.*, 2010 ▶, 2011*c* ▶). The fibrillar morphology agrees with a β-helix with a period of 5.72 nm (Accardo *et al.*, 2011*c* ▶).

**Figure 9:**
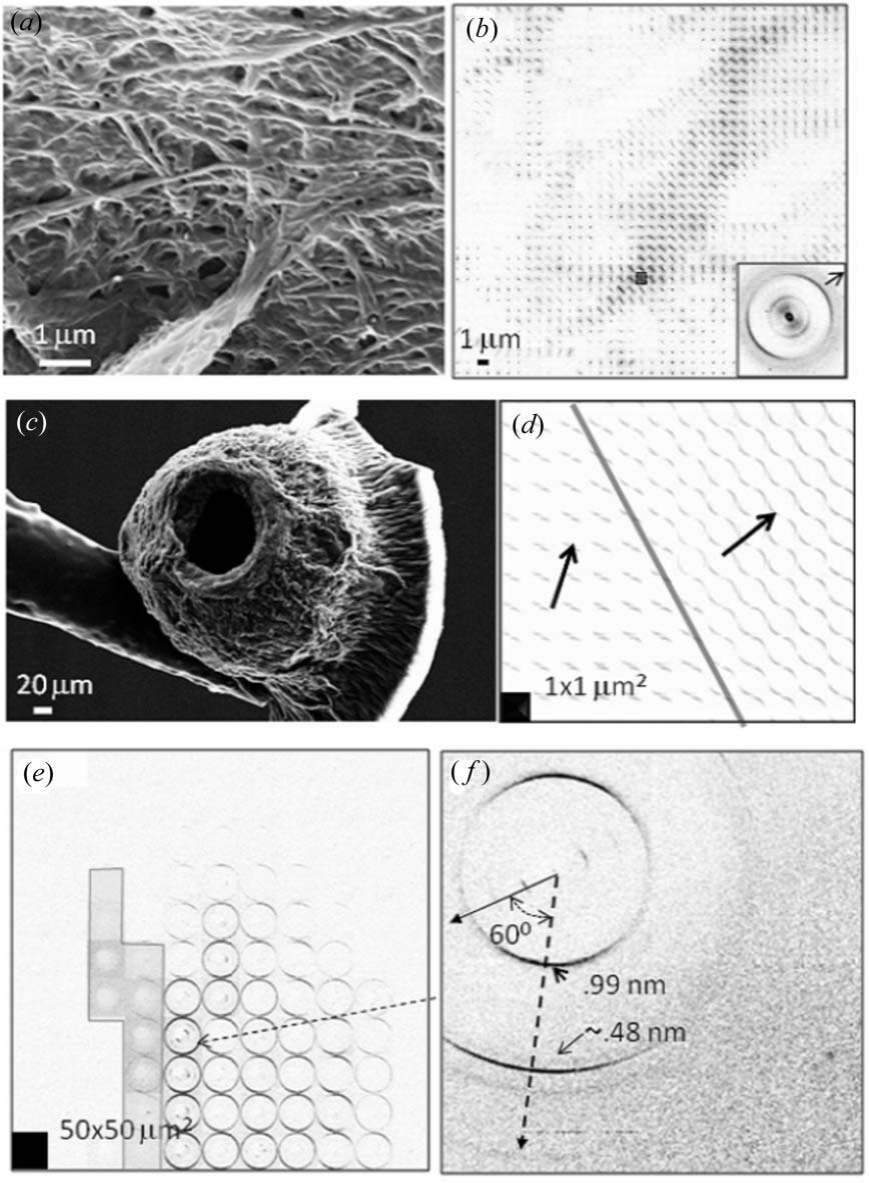
(*a*) FESEM image of fibrillar Ac-NFGAIL morphology (Lakshmanan *et al.*, 2013 ▶). (*b*) Raster-scan image of fibrillar morphology obtained with a 200 nm X-ray beam. The inset shows a single WAXS pattern from the position of the rectangle in (*b*). The fibre axis is indicated by an arrow. [Adapted from Lakshmanan *et al.* (2013 ▶).] (*c*) SEM image of hollow Ac-KLVFFAE residue glued to a glass capillary (Lakshmanan *et al.*, 2013 ▶). (*d*) Raster-scan image of bulk residue revealing two domains with homogeneous fibre orientation (arrow) in each domain defined by the orientation of the 0.47 nm β-sheet peak. (*e*) Raster-scan of residue glued to the capillary (contours indicated) with oriented patterns from the pinning zone at the hole [see also (*c*)]. (*f*) Oriented pattern with two principal reciprocal lattice lines at an angle of ∼60°. The first and second (weak) cross-β peaks are indicated. [Adapted from Lakshmanan *et al.* (2013 ▶).]

The nanofibrillar morphology of the human islet core sequence (Ac-NFGAIL) is revealed by SEM and by X-ray nanobeam raster scans (Lakshmanan *et al.*, 2013 ▶) [Figs. 9(*a*) and 9(*b*) ▶]. A highly oriented fibre diffraction pattern of the cross-β structure can be resolved from a zone with few fibrils [Fig. 9(*b*) ▶ and inset]. Shearing effects during pinning of the droplet can result in new structures as shown for β-amyloid (16–22) (Ac-KLVFFAE) (Lakshmanan *et al.*, 2013 ▶) [Figs. 9(*c*)–9(*f*) ▶]. Domains of disordered lamellar cross-β slabs are found in the bulk residue. The structure observed in the pinning area does not, however, correspond to an orthogonal cross-β pattern [*e.g.* Fig. 9(*b*) ▶, inset]. The angle of about 60° between the major reciprocal lattice directions has been attributed to tilted cross-β slabs (Lakshmanan *et al.*, 2013 ▶). Conformational mixtures, such as different β-sheet morphologies, can be unravelled on superhydrophobic and superhydrophilic substrates by a combination of spectroscopic and SAXS/WAXS techniques. Indeed, the interaction of β-amyloid (1–42; 25–35) with phospholipids simulating neuronal membranes was explored by a combination of µFTIR and µSAXS/WAXS (Accardo *et al.*, 2014 ▶).

### 3.3. Biological microsystems: cells and subcellular components   

Concentration, aggregation and assembly on SHSs can be extended also to living cells and subcellular components providing the possibility of probing highly oriented zones in an evaporating droplet or residue. The low contact forces to the surface may be critical for enhancing interactions between the biological objects.

#### 3.3.1. Cells   

Textured surfaces of cells play an important role in site-selective immobilization; wettability, charge and roughness are suitable for cell attachment, whereas hydrophobic and smooth surfaces tend to prevent cells from adhering and growing (Ishizaki *et al.*, 2010 ▶). In an original and effective way it was demonstrated that a vertically aligned silicon nanopatterned device with very low wettability promotes three-dimensional neuronal growth and differentiation (Limongi *et al.*, 2013 ▶) opening interesting scenarios in the development of implantable neuroprosthetic devices or in tissue regeneration therapies. This three-dimensional superhydrophobic scaffold also possesses adequate stability which enables neuronal cells growth and at the same time could allow carrying raster-scan µSAXS and SR tomography for identifying synapses and clarifying the structure of complex neuronal networks.

#### 3.3.2. Exosomes   

**Figure 10:**
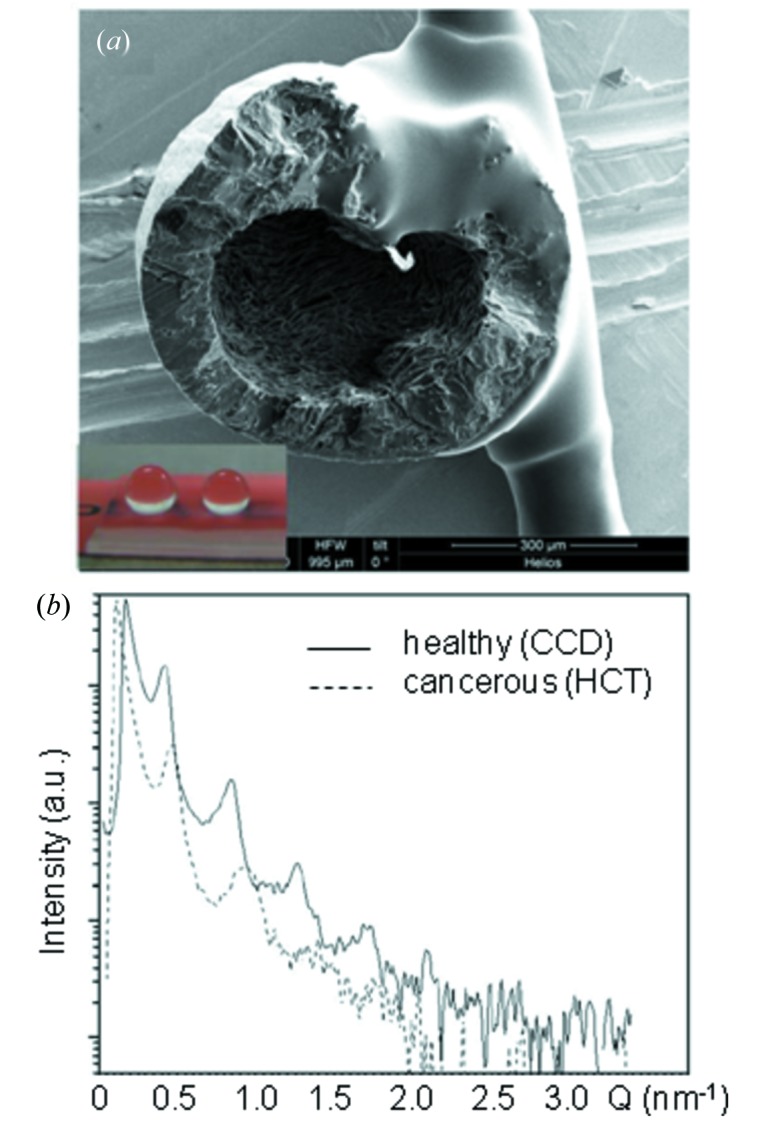
(*a*) SEM image of an exosomes residue from a cancerous (HCT) colon cell line dried on a PMMA SHS revealing a lamellar morphology. (*b*) SAXS data showing different lamellar periodicities for exosome residues derived from HCT and CCD (healthy) colon cell lines. [Adapted from Accardo *et al.* (2013*b* ▶).]

Exosomes are cell-derived vesicles (40–100 nm in diameter) which play a key role in processes such as coagulation, intercellular signalling and waste management (Lai & Breakefield X, 2012 ▶). It has now been shown that exosome residues on PMMA SHSs, coming from healthy (CCD) and cancerous (HCT) colon cell lines, can be discriminated by µSAXS probing (Accardo *et al.*, 2013*b* ▶) [Figs. 10(*a*) and 10(*b*) ▶]. Indeed, the observed lamellar morphology observed by µSAXS (Fig. 10*b* ▶) reveals significant differences in the number of orders, their periodicities (*L*) and peak broadening (*e.g.* *L*_CCD_ = 13.5 ± 0.5 nm and *L*_HCT_ = 15.0 ± 0.5 nm). The sensitivity of the experiment can be attributed to an alignment of the lamellar residues (Fig. 10*a* ▶). This has also allowed HCT/CCD residues to be differentiated with a laboratory SAXS set-up, although with a larger beam size, allowing routine probing for the signature of exosomes scattering prior to a SR experiment.

## 4. Conclusions and outlook   

Droplets on SHSs correspond to quasi contact-free sample environments. Indeed, the easy diffusion of gases across the liquid/gas interface could be used for studying effects of environmental agents on reaction equilibriums. The liquid/air interface is also of practical interest as it allows avoiding contributions of cell walls to X-ray absorption and scattering. Techniques for controlling droplet volumes and for rapid mixing of droplets have been demonstrated. Integration into practical devices is, however, still lacking. Indeed, the SHEWOD technology is an interesting alternative to stopped-flow mixing devices for probing chemical and biological kinetics at sub-millisecond timescales. The ability to control droplet volumes of a few microlitres and less can be used for initiating and probing of biological processes without confining walls.

Solution concentration by at least an order of magnitude allows concentration-dependent processes such as nucleation, aggregation and assembly to be studied. In general, these processes occur at liquid/air interfaces or during pinning resulting in ordering effects enhancing information obtainable by micro/nanobeam SAXS/WAXS probes. One can expect that complementary optical, imaging and spectroscopy techniques will be increasingly used for studying such processes.
